# The Phase II Study of Panitumumab in Chemotherapy-Naïve Frail or Elderly Patients with *RAS* Wild-type Colorectal Cancer: OGSG 1602 Final Results

**DOI:** 10.1093/oncolo/oyac145

**Published:** 2022-08-10

**Authors:** Tetsuji Terazawa, Takeshi Kato, Masahiro Goto, Katsuya Ohta, Hironaga Satake, Shingo Noura, Yoshinori Kagawa, Hisato Kawakami, Hiroko Hasegawa, Kazuhiro Yanagihara, Tatsushi Shingai, Ken Nakata, Masahito Kotaka, Masayuki Hiraki, Ken Konishi, Shiro Nakae, Daisuke Sakai, Yukinori Kurokawa, Toshio Shimokawa, Toshimasa Tsujinaka, Taroh Satoh

**Affiliations:** Cancer Chemotherapy Center, Osaka Medical and Pharmaceutical University, Takatsuki-City, Japan; Department of Gastroenterological Surgery, National Hospital Organization Osaka National Hospital, Higashiosaka, Japan; Cancer Chemotherapy Center, Osaka Medical and Pharmaceutical University, Takatsuki-City, Japan; Department of Gastroenterological Surgery, Higashiosaka City Medical Center, Osaka, Japan; Department of Medical Oncology, Kobe City Medical Center General Hospital, Kobe, Japan; Department of Gastroenterological Surgery, Osaka Rosai Hospital, Sakai, Japan; Department of Gastroenterological Surgery, Toyonaka Municipal Hospital, Toyonaka, Japan; Department of Surgery, Kansai Rosai Hospital, Amagasaki, Japan; Department of Medical Oncology, Kindai University Faculty of Medicine, Osakasayama, Japan; Department of Gastroenterology and Hepatology, National Hospital Organization Osaka National Hospital, Osaka, Japan; Department of Medical Oncology, Kansai Electric Power Hospital, Osaka, Japan; Department of Surgery, Saiseikai Senri Hospital, Suita, Japan; Department of Surgery, Sakai City Medical Center, Sakai, Japan; Gastrointestinal Cancer Center, Sano Hospital, Kobe, Japan; Department of Surgery, Itami City Hospital, Itami, Japan; Department of Surgery, Hyogo Prefectural Nishinomiya Hospital, Nishinomiya, Hyogo, Japan; Department of Medical Oncology, Mimihara General Hospital, Sakai, Japan; Department of Frontier Science for Cancer and Chemotherapy, Osaka University Graduate School of Medicine, Suita, Japan; Department of Gastroenterological Surgery, Osaka University Graduate School of Medicine, Osaka, Japan; Department of Medical Data Science, Graduate School of Medicine, Wakayama Medical University, Suita, Japan; Cancer Center, Izumi City General Hospital, Izumi, Japan; Department of Frontier Science for Cancer and Chemotherapy, Osaka University Graduate School of Medicine, Suita, Japan

**Keywords:** colorectal cancer, frail patient, elderly patients, panitumumab

## Abstract

**Background:**

We previously reported the response rate of a phase II OGSG1602 study on panitumumab in chemotherapy-naive frail or elderly patients with *RAS* wild-type unresectable colorectal cancer (CRC) [Terazawa T, Kato T, Goto M, et al. *Oncologist*. 2021;26(1):17]. Herein, we report a survival analysis.

**Methods:**

Patients aged ≥65 years and considered unsuitable for intensive chemotherapy or aged ≥76 years were enrolled. Primary tumors located from the cecum to the transverse colon were considered right-sided tumors (RSTs); those located from the splenic flexure to the rectum were considered left-sided tumors (LSTs).

**Results:**

Among the 36 enrolled patients, 34 were included in the efficacy analysis, with 26 and 8 having LSTs and RSTs, respectively. The median progression-free survival (PFS) and overall survival (OS) were 6.0 [95% CI, 5.4-10.0] and 17.5 months (95% CI, 13.8-24.3), respectively. Although no significant differences existed in PFS between patients with LST and RST {6.6 (95% CI, 5.4-11.5) vs. 4.9 months [95% CI, 1.9-not available (NA), *P* = .120]}, there were significant differences in OS [19.3 (95% CI, 14.2-NA) vs.12.3 months (95% CI, 9.9-NA), *P* = .043].

**Conclusion:**

Panitumumab showed favorable OS in frail or elderly patients with *RAS* wild-type CRC and no prior exposure to chemotherapy. Panitumumab may be optimal for patients with LSTs (UMIN Clinical Trials Registry Number UMIN000024528).

## Teaser

This article presents a survival analysis of the phase II OGSG1602 trial on panitumumab in chemotherapy-naive frail or elderly patients with RAS wild-type unresectable colorectal cancer.

**Lessons Learned**

- The final analysis of OGSG1602 confirmed the efficacy of panitumumab as a first-line treatment for PFS and OS in frail or elderly patients with *RAS* wild-type unresectable colorectal cancer.
- In particular, panitumumab monotherapy as the first-line treatment may be optimal for patients with left-sided tumors.
- Patients receiving panitumumab who achieved early tumor shrinkage or depth of response showed consistently greater improvements in PFS and OS than those who did not.

## Discussion

In the previously published primary analysis, OGSG1602 met its primary endpoint of 76.5% disease control rate (DCR) with a 50% response rate (RR).^1^ In this final analysis, panitumumab showed favorable survival results as the first-line treatment for patients with *RAS* wild-type colorectal cancer who were ineligible for intensive chemotherapy, with a median OS and PFS of 17.5 months and 6.0 months, respectively. We also assessed survival according to the primary tumor location. Our data confirmed that panitumumab monotherapy as the first-line treatment was optimal for patients with left-sided tumors but not recommended for those with right-sided tumors (median PFS: 6.6 months vs. 4.9 months and median OS: 19.3 months vs. 12.3 months, respectively), which was in line with our previous report in that the RR of patients with left-sided tumors and right-sided tumors was 65.4% and 0.0%, respectively.^1^

Interestingly, a retrospective analysis of the NCIC CTG CO.17, which compared cetuximab with BSC, also reported that cetuximab significantly improved PFS in patients with *KRAS* wild-type left-sided tumors (median: 5.4 vs. 1.8 months), but not in those with right-sided tumors (median: 1.9 vs. 1.9 months).^2^ Left-sided tumor is derived from the embryonic hindgut, whereas a right-sided tumor is derived from the embryonic midgut. Notably, right-sided tumors are more frequently characterized by a host of adverse prognostic factors, including *BRAF* mutation, microsatellite instability-high, hypermutation, serrated pathway signature positivity, and mucinous histology, while left-sided tumors more frequently possess gene expression profiles characteristic of an EGFR inhibitor-sensitive phenotype.^3-5^ These molecular differences manifest as differential clinical behavior, with right-sided tumors typically having a poor prognosis.

In conclusion, the final analysis of OGSG1602 confirmed the efficacy of panitumumab as a first-line treatment for PFS and OS in frail or elderly patients with *RAS* wild-type unresectable colorectal cancer. In particular, panitumumab monotherapy as the first-line treatment may be optimal for patients with left-sided tumors. Therefore, panitumumab offers a new option for frail or elderly patients based on the tumor *RAS* status and sidedness.

## Trial Information

**Table:** 

Disease	Colorectal cancer: *RAS* wild type
Stage of disease/treatment	Metastatic/advanced
Prior therapy	None
Type of study	Phase II study
Primary endpoint	Disease-control rate
Secondary endpoints	Response rate, progression-free survival, time to treatment failure, toxicity.
Investigator’s analysis	Active and should be pursued further

## Additional Details of Endpoints or Study Design

### Patients, Treatment, and Study Design

Details of this study have been described previously.^1^ OGSG1602 was an open-label, one-arm, phase II study conducted at 14 medical centers, university hospitals, and general hospitals in Japan.

The eligibility criteria were as follows: patients aged ≥76 or ≥65 years who were considered ineligible for intensive chemotherapy by the treating investigator, histologically or cytologically conﬁrmed carcinoma of the colon/rectum, *RAS* wild-type, evidence of metastases, at least one measurable lesion per Response Evaluation Criteria in Solid Tumors (version 1.1), creatinine clearance of at least 30 mL/min, a life expectancy of 3 months or longer at enrollment, no primary chemotherapy, and no prior anti-EGFR antibody therapy.

Panitumumab 6 mg/kg intravenous infusion was administered every 2 weeks. Patients received treatment until the appearance of progressive disease, unacceptable toxicities, patient withdrawal, physician’s decision, or planned conversion surgery with intended curative resection. The patients were withdrawn from the study when treatment could not be started within 28 days.

The primary endpoint was disease control rate (DCR), defined as the proportion of the best overall response from either complete response (CR), partial response (PR), or stable disease. We set the primary endpoint as DCR considering the features of the standard treatment, capecitabine plus bevacizumab, which had a favorable DCR as compared with RR. The PR was not confirmed. The DCR was also assessed by an independent review committee. Disease re-assessment was performed using contrast-enhanced computed tomography every 8 weeks. The secondary endpoints were as follows: OS, defined as the time from enrollment to death from any cause; PFS, deﬁned as the time from enrollment to disease progression or death from any cause; RR, defined as the proportion of best overall response of CR or PR; time-to-treatment failure (TTF), defined as the time from enrollment to discontinuation of treatment for any reason, including disease progression, treatment toxicity, and death; and the incidence of grade 3/4 toxicities according to the Common Terminology Criteria for Adverse Events version 4.0.

### Statistical Analysis

The null hypothesis was 45%, and the alternative hypothesis was 70%, which was assessed by the Clopper–Pearson method using an exact *P*-value of .05 and a power of 0.90. Given that 33 patients were required, the total sample size was set as 36 to account for drop-outs. TTF, PFS, and OS were estimated using the Kaplan–Meier method. An exact 95% CI was estimated for stratified odds ratios for DCR and RR.

Post-hoc analyses were carried out to examine the effect of primary tumor location, depth of response, early tumor shrinkage, and impact of hypomagnesemia on efficacy, including PFS and OS. Primary tumors located from the cecum to the transverse colon were considered right-sided tumors, while those located from the splenic flexure to the rectum were considered left-sided tumors. Early tumor shrinkage was defined as a tumor reduction of 20% or more at week 8 compared to that at baseline; depth of response was defined as the percentage of tumor shrinkage at nadir or progression; and hypomagnesemia was determined as grade 2 or higher. In addition, the time-dependent receiver operating characteristic (ROC) curve was used to investigate the relationship between early tumor shrinkage/depth of response and OS. The Youden index, defined as the maximum vertical distance between the ROC curve and the diagonal or chance line, was used to determine the optimal cut-off point.^6^ Landmark analysis of a subgroup of patients with hypomagnesemia (the highest grade of 0–1 vs. 2 or higher), which examines the relationship between the number of cycles and the hazard ratio (HR) of OS at each cycle, was also used to investigate the predictive value of hypomagnesemia. For landmark analysis, we chose time points as a cycle (0–10) and estimated HR at each landmark time point.

All statistical analyses were conducted at the OGSG Data Center. Statistical analyses were conducted using R version 3.5.1 (The R Foundation for Statistical Computing, Vienna, Austria; https://www.R-project.org/).

## Drug Information

**Table:** 

Generic/working name	Panitumumab
**Company name**	Takeda Pharmaceutical Company Limited.
**Drug type**	Antibody
Drug class	EGFR
Dose	6 mg/kg
**Route**	Intravenous infusion
Schedule of administration	Every 2weeks

## Patient Characteristics

**Table:** 

Number of patients, male	20
Number of patients, female	16
**Stage**	IV/recurrence, 20/16
Age, median (range)	81 (67–88) years
Number of prior systemic therapies, median (range)	None
Performance status: ECOG	0: 18
	1: 15
	2: 2
	3: 1
	4: 0
Cancer types or histologic subtypes	Tubular adenocarcinoma, 31; poorly differentiated adenocarcinoma, 2

## Primary Assessment Method

**Table:** 

Title	Effectiveness in all patients
Number of patients screened	36
Number of patients enrolled	36
Number of patients evaluable for toxicity	36
Number of patients evaluated for efficacy	34
Evaluation method	RECIST 1.1
Response assessment, CR	3 (8.8%)
Response assessment, PR	14 (41.2%)
Response assessment, SD	9 (26.5%)
Response assessment, PD	6 (17.6%)
Median duration assessment, PFS	6 months (CI: 5.0-10.4)
Median duration assessment, TTP	4.5 months (CI: 3.1-5.8)
Median Duration Assessment, OS	17.5 months (CI: 13.8-24.3)

## Secondary Assessment Method

**Table:** 

Title	Effectiveness in left-sided tumors
Number of patients evaluated for efficacy	26
Evaluation method	RECIST 1.1
Response assessment, CR	3 (11.5%)
Response assessment, PR	14 (53.8%)
Response assessment, SD	4 (15.4%)
Response assessment, PD	3 (11.5%)
Median duration assessment, PFS	8.6 months (CI:5.4-11.5)
Median duration assessment, TTP	4.5 months (CI: 3.1-5.8)
Median duration assessment, OS	19.3 months (CI: 14.2-not available)

## Outcome Notes

### Update of Treatment Delivery

In total, 36 patients were enrolled in this study between February 2017 and August 2018; the median age of patients was 81 (range: 67-88) years.^1^ The median number of cycles was 8 (range: 1-16). The final median TTF was 4.5 months (95% CI, 3.1-5.8). Furthermore, 11 (30.6%) and 4 patients (11.1%) had their doses reduced by one and two levels, respectively. The reasons for dose reduction were as follows: rash (*n* = 5), fatigue (*n* = 5), hypomagnesemia (*n* = 2), poor performance status (*n* = 2), stomatitis (*n* = 1), paronychia (*n* = 1), and physician’s discretion (*n* = 1). The reasons for discontinuation are summarized in Table 1. The median follow-up period was 17.0 months from enrollment.

**Table 1.** Reasons for discontinuation (*n* = 36)

Reason	*n*
Disease progression	18
**Toxicities**	5
Paronychia G3, stomatitis G1, fatigue G1	1
Rash G2, fatigue G2, stomatitis G1, hypomagnesemia G2	1
Sarcopenia	1
Hypomagnesemia G2	1
Infusion reaction G3	1
**Could not start the next cycle within four weeks**	5
Hypomagnesemia	2
Rash	2
Hypomagnesemia, rash	1
**Patients wish (because of complete response)**	2
Conversion surgery	2
Radiation therapy	1
Other	3

Abbreviation: G, grade (according to CTCAE Ver 4.0).

### Efficacy

Among the 34 patients who were included in the analysis of efficacy,^1^ 15 (44.1%) achieved early tumor shrinkage, while 19 (55.9%) did not. All patients who achieved early tumor shrinkage had left-sided tumors.

**Figure 1.**
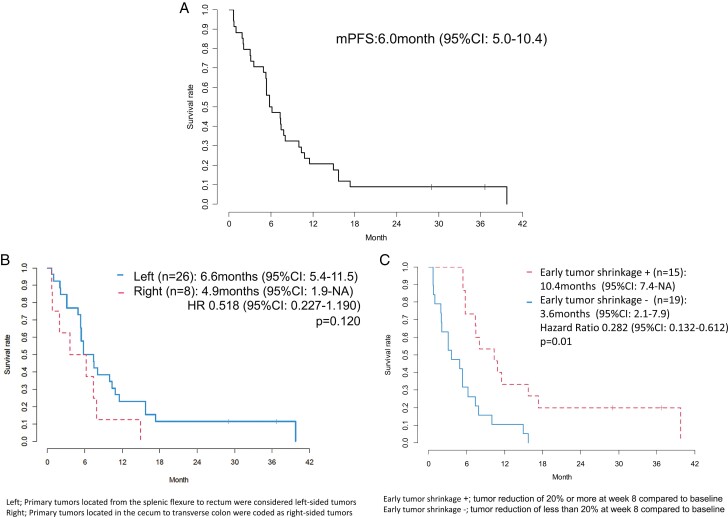
**(A)** Progression-free survival. **(B)** Progression-free survival by tumor location (*n* = 34; Left [*n* = 26]: primary tumors located from the splenic flexure to the rectum were coded as left-sided tumors; right [*n* = 8]: primary tumors located in the cecum to the transverse colon were coded as right-sided tumors). **(C)** Progression-free survival by early tumor shrinkage (*n* = 34; early tumor shrinkage + [*n* = 15]: tumor reduction of 20% or more at week 8 compared to baseline; early tumor shrinkage – [*n* = 19]: tumor reduction of less than 20% at week 8 compared to baseline).

The median PFS was 6.0 months (95% CI, 5.4-10.0; Fig. 1a). A PFS benefit was observed in patients with left-sided tumors as against right-sided tumors (HR: 0.518; 95% CI, 0.227-1.190; *P* = .120; Fig. 1b), with a median PFS of 6.6 (95% CI, 5.4-11.5) vs. 4.9 months [95% CI, 1.9-not available (NA)], respectively. A significant improvement in PFS was observed in patients with positive as against negative early tumor shrinkage (HR: 0.282; 95% CI, 0.132-0.612; *P* = .001; Fig. 1c), with a median PFS of 10.4 (95% CI, 7.4-NA) vs. 3.6 months (95% CI, 2.1-7.9), respectively.

**Figure 2.**
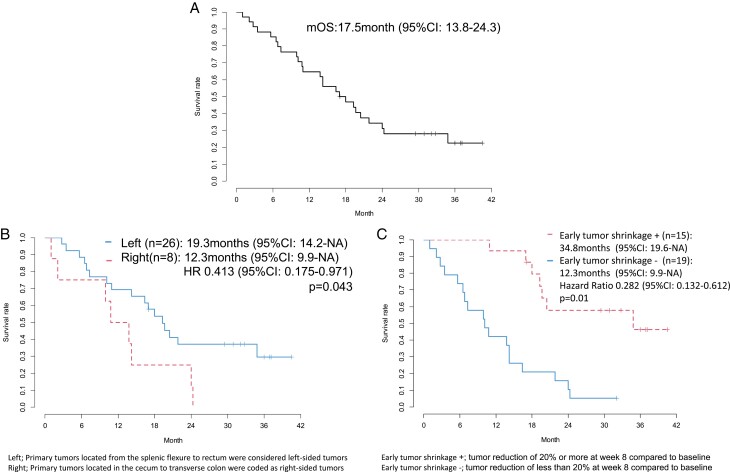
**(A)** Overall survival (*n* = 34). **(B)** Overall survival by primary tumor location (*n* = 34; Left [*n* = 26]: primary tumors located from the splenic flexure to the rectum were corded left-sided tumors; Right [*n* = 8]: primary tumors located in the cecum to the transverse colon were coded as right-sided tumors. **(C)** Overall survival by early tumor shrinkage (*n* = 34; early tumor shrinkage + [*n* = 15]: tumor reduction of 20% or more at week 8 compared to baseline; early tumor shrinkage − [*n* = 19]: tumor reduction of less than 20% at week 8 compared to baseline).

**Figure 3.**
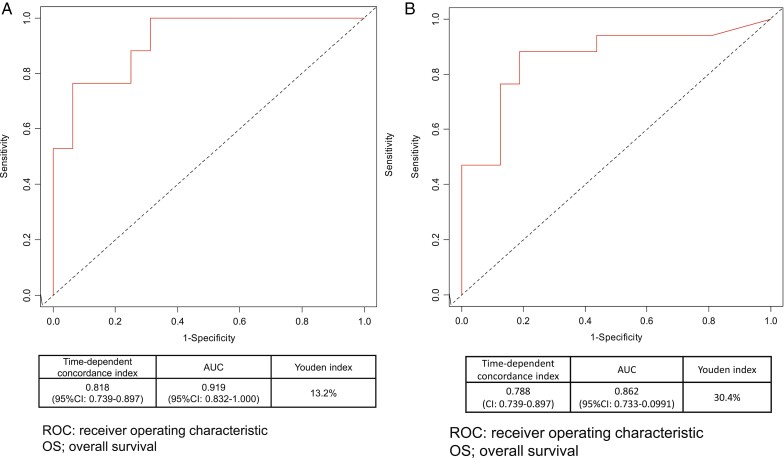
Time-dependent ROC curve on OS by tumor shrinkage **(A)** and by depth of response **(B)** (*n* = 34). Abbreviations: ROC, receiver operating characteristic; OS, overall survival.

The median OS was 17.5 months (95% CI, 13.8-24.3; Fig. 2a). A statistically significant OS benefit was observed in patients with left-sided tumors as against those with right-sided tumors (HR: 0.413; 95% CI, 0.175-0.971; *P* = .043; Fig. 2b), with a median OS of 19.3 (95% CI, 14.2-NA) vs. 12.3 months (95% CI, 9.9-NA), respectively. Similar findings were observed in patients with positive as against those with negative early tumor shrinkage (HR: 0.184; 95% CI, 0.072-0.472; *P* < .001; Fig. 2c), with a median OS of 34.8 (95% CI, 19.6-NA) versus 12.3 months (95% CI, 9.9-NA), respectively. Based on the results of the time-dependent area under the curve, which was estimated to examine the consistency of the treatment effects of early tumor shrinkage and depth of response on OS, the time-dependent concordance indices were 0.818 (95% CI, 0.739-0.897) and 0.919 (95% CI, 0.832-1.000) in early tumor shrinkage (Fig. 3a), and 0.788 (95% CI, 0.739-0.897) and 0.863 (95% CI, 0.733-0.0991) in depth of response, respectively (Fig. 3b). The Youden index, which was examined to determine the optimal cut-off point, was 13.2% for early tumor shrinkage and 30.4% for depth of response. Thus, our data demonstrated that early tumor shrinkage and depth of response showed predictive values for OS, with the cut-off early tumor shrinkage and depth of response in this trial being 13.2% and 30.4%, respectively.

**Figure 4.**
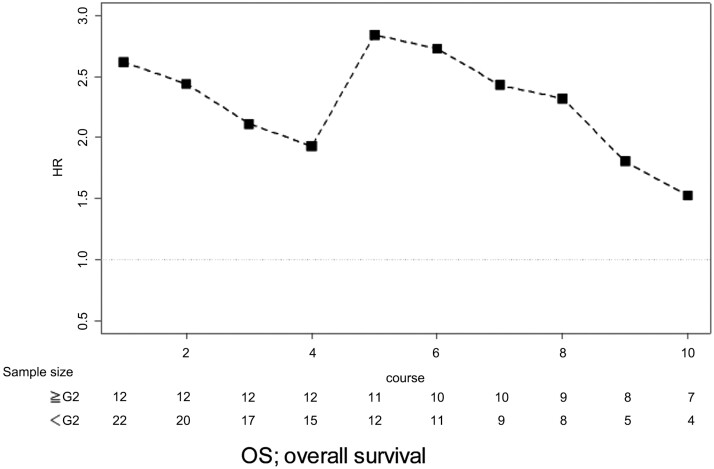
Landmark analysis on OS regarding hypomagnesemia (*n* = 34). Abbreviation: OS, overall survival.

The landmarked analysis of subgroups of patients with hypomagnesemia (grade 0-1, *n* = 12 vs. 2 or higher, *n* = 22) showed that the value of HR was approximately 2.0-2.5 for 1-8 cycles and the bottom of HR was 1.5 (Fig. 4), with the median cycle number of 10.5 [interquartile range (IQR): 7.75-11.25] in patients with grade 2 or higher hypomagnesemia and 5.5 (IQR: 3.0-8.0) in patients with less than grade 2 hypomagnesemia, respectively.

### Toxicities and Subsequent Therapy

The updated safety data did not differ from that of our first report,^1^ and there were no treatment-related deaths.^1^ In brief, the major grade 3 or 4 toxicities in all 36 patients were rash (*n* = 6, 17%), hypomagnesemia (*n* = 4, 11%), fatigue (*n* = 3, 8%), paronychia (*n* = 2, 6%), and hyponatremia (*n* = 2, 6%). The results of the subsequent therapies are summarized in Table 2. After this protocol treatment in the 34 eligible patients, 12 (35.3%) received best supportive care (BSC), followed by a fluorouracil-based regimen (*n* = 4), oxaliplatin-based regimen (*n* = 4), panitumumab monotherapy (*n* = 4), curative radiation therapy (*n* = 4), surgery (*n* = 3), irinotecan-based regimen (*n* = 1), and other therapies (*n* = 1).

**Table 2.** Subsequent therapy (*n* = 34)

Therapy	*n*
Best supported care	12
Oxaliplatin based regimen	4
Curative radiation therapy	4
Fluorouracil based regimen	4
Panitumumab monotherapy	4
Surgery	3
Irinotecan based regimen	1
Palliative radiation therapy	1
Other therapy	1

## Secondary Assessment Method

**Table:** 

Title	Efficacy in right-sided tumors
Number of patients evaluated for efficacy	8
Evaluation method	RECIST 1.1
Response assessment, CR	0 (0%)
Response assessment, PR	0 (0%)
Response assessment, SD	5 (62.5%)
Response assessment, PD	3 (37.5%)
Median duration assessment, PFS	4.9 months (CI: 1.9-not available)
Median duration assessment, OS	12.3 months (CI: 9.9-not available)

## Assessment, Analysis, and Discussion

**Table:** 

Completion	Study completed
Investigator’s assessment	Active and should be pursued further

Advanced colorectal cancer is the second most common cause of cancer-related deaths worldwide after lung cancer.^7^ The development of new cytotoxic drugs has increased the median survival time of patients with metastatic colorectal cancer from 8 to approximately 30 months, over the past two decades.^8-10^ In contrast, frail or elderly patients are often excluded from randomized trials or represent a minority of enrolled patients,^11^ despite the high prevalence of metastatic colorectal cancer in this population.^12^ Approximately 60% of patients newly diagnosed with cancer are 65 years or older, making this the commonest population group seen in most oncology practices.^12^ In addition, given that elderly or frail patients are more likely to present with a decline in organ function and comorbidities at diagnosis, they are at a higher risk of adverse events than fit patients.^13,14^ Therefore, several trials targeting frail or elderly patients with metastatic colorectal cancer have been undertaken.^15,16^ Less intensive regimens, such as capecitabine plus bevacizumab, or reduced-dose oxaliplatin plus 5-fluorouracil, were more optimal for frail and elderly patients.^15,16^ Furthermore, anti-epidermal growth factor receptor (EGFR) therapy is an attractive option for frail or elderly patients with *RAS* wild-type metastatic colorectal cancer.^17^

Activating *KRAS* and *NRAS* mutations occur in 30-50% and 3-5% of patients, respectively.^18,19^ Given that the presence of any activating *RAS* mutations (*KRAS* exon 3 or 4 and *NRAS* exons 1, 2, 3, or 4) predicts a lack of benefit of panitumumab or cetuximab in the first-line treatment of metastatic colorectal cancer, upfront determination of an all-*RAS* mutational analysis beyond KRAS exon 2 to include KRAS exons 2 through 4 and NRAS exons 1 through 4 has been recommended.^18,19^

Frail and elderly patients showed favorable results in two recent prospective trials in which panitumumab was administered in patients with KRAS wild-type as first-line therapy and in those with RAS wild-type as second-line therapy.^20,21^ The efficacy and safety data of EGFR-inhibiting monoclonal antibodies as first-line therapy in patients with all-RAS wild-types have not been extensively investigated. To the best of our knowledge, ours is the first trial of panitumumab therapy for frail or elderly patients specified for first-line and with *RAS* wild-type, providing baseline information for the selection of less intensive treatments.^1^

In terms of primary tumor location, which is considered a predictive biomarker of anti-EGFR antibody plus chemotherapy,^22,23^ anti-EGFR antibody plus chemotherapy is preferred as a first-line treatment option for patients with left-sided tumors, while patients with right-sided tumors generally appear to benefit less from this treatment.^22,23^ Our first report of panitumumab monotherapy also showed significantly higher RR in patients with left-sided tumors (*n* = 26) than in those with right-sided tumors (*n* = 8) (65.4% vs. 0.0%; *P* = .003).^1^ To date, no studies have investigated the survival data of tumor sidedness in panitumumab monotherapy for *RAS* wild-type colorectal cancer. In this final analysis, panitumumab showed favorable survival results as the first-line treatment for patients with *RAS* wild-type who were ineligible for intensive chemotherapy. In addition, our data confirmed that panitumumab monotherapy as first-line treatment was optimal for patients with left-sided tumors but not recommended for those with right-sided tumors.

We also examined the other biomarkers such as early tumor shrinkage and depth of response have potential predictive importance with anti-EGFR antibody plus chemotherapy in metastatic colorectal cancer.^24^ Early tumor shrinkage appeared to be associated with improved PFS and OS in panitumumab monotherapy, as previously observed in anti-EGFR antibody plus chemotherapy.^24^ Furthermore, the time-dependent ROC curve suggested that early tumor shrinkage and depth of response could be predictive factors, which is consistent with previous reports of anti-EGFR therapy combined with cytotoxic agents.^25,26^ In addition, according to the Youden index, which was intended to determine the optimal cut-off point,^6^ the cut-off value of tumor reduction at first evaluation was 13.2% for early tumor shrinkage, while that of tumor reduction as the best response was 30.4% for depth of response. We pre-defined early tumor shrinkage as more than 20% of tumor reduction at 8 weeks from baseline, which was considered reasonable from our results. We also examined the predictive value of hypomagnesemia on efficacy using landmark analysis. The landmark analysis showed a HR of more than 1.5 for OS in the patients with hypomagnesemia grade 2 or more at each cycle compared; however, the first appearance of grade 2 or higher hypomagnesemia occurred at 4 cycles or later regardless of the median cycle number of 5.5 in patients with less than grade 2 hypomagnesemia. Considering that our data showed severe hypomagnesemia occurred after repeated-administrations, hypomagnesemia appeared to be unsuitable as a predictive marker.

Capecitabine plus bevacizumab is widely accepted as the standard treatment for patients who are ineligible for intensive chemotherapy.^15,17^ The AVEX trial demonstrated that the PFS and OS of capecitabine plus bevacizumab were 9.1 and 20.7 months, respectively, despite a 19% RR and 4% grade 5 adverse events.^15^ The survival outcome of the AVEX trial seemed better than that of our trial, probably because more than half of the patients in our trial were over the age of 80. Nevertheless, patients with left-sided tumors showed a median PFS of 6.6 months and an OS of 19.3 months, which was comparable with the results of the capecitabine plus bevacizumab regimen. Considering the favorable efficacy and tolerability, panitumumab monotherapy may be a reasonable choice as a first-line therapy for frail or elderly patients, especially those with left-sided tumors.

This study has several limitations. First, given that OGSG1602 is a single-arm phase II trial, the findings of this trial could not be conclusive; however, our trial confirmed the findings from previous reports of anti-EGFR antibody plus chemotherapy,^24^ underscoring the importance of sidedness in determining the predictive value of panitumumab.^22,23^ Second, the presence of *BRAF* mutation or microsatellite instability was not investigated, since those were not approved at the beginning of this study in Japan. In contrast, since *BRAF* mutation or microsatellite instability-high mainly occurs in patients with right-sided tumors,^3-5^ also suggests the importance of sidedness. Finally, formal geriatric and co-morbidity assessments were not performed as part of the trial.

In conclusion, the final analysis of OGSG1602 confirmed the efficacy of panitumumab as a first-line treatment for PFS and OS in frail or elderly patients with *RAS* wild-type unresectable colorectal cancer. In particular, panitumumab monotherapy as the first-line treatment may be optimal for patients with left-sided tumors. Patients receiving panitumumab who achieved early tumor shrinkage or depth of response showed consistently greater improvements in PFS and OS than those who did not. Therefore, panitumumab offers a new option for frail or elderly patients based on the tumor *RAS* status and sidedness.

## Data Availability

The data underlying this article were provided by OGSG and Takeda under license. Data will be shared on request to the corresponding author with the permission of OGSG and Takeda.
